# DNA Methyltransferases in Malar Melasma and Their Modification by Sunscreen in Combination with 4% Niacinamide, 0.05% Retinoic Acid, or Placebo

**DOI:** 10.1155/2019/9068314

**Published:** 2019-04-22

**Authors:** Andres Eduardo Campuzano-García, Bertha Torres-Alvarez, Diana Hernández-Blanco, Cornelia Fuentes-Ahumada, Juan Diego Cortés-García, Juan Pablo Castanedo-Cázares

**Affiliations:** Dermatology Department, Hospital Central Dr. Ignacio Morones Prieto, Universidad Autónoma de San Luis Potosí, Mexico

## Abstract

**Background:**

Malar melasma has a chronic and recurrent character that may be related to epigenetic changes.

**Objective:**

To recognize the expression and DNA methylation of DNA methyltransferases (DNMTs) in malar melasma and perilesional skin, as well as the changes in DNMTs after their treatment with sunscreen in combination with 4% niacinamide, 0.05% retinoic acid, or placebo.

**Methods:**

Thirty female patients were clinically evaluated for the expression of DNMT1 and DNMT3b using real-time PCR and immunofluorescence. These initial results were compared to results after eight weeks of treatment with sunscreen in combination with niacinamide, retinoic acid, or placebo.

**Results:**

The relative expression of DNMT1 was significantly elevated in melasma compared with unaffected skin in all subjects, indicating DNA hypermethylation. After treatment, it was decreased in all groups: niacinamide (7 versus 1; p<0.01), retinoic acid (7 versus 2; p<0.05), and placebo (7 versus 3; p<0.05), which correlates with clinical improvement. DNMT3b was not overexpressed in lesional skin but reduced in all groups.

**Conclusions:**

We found DNA hypermethylation in melasma lesions. Environmental factors such as solar radiation may induce cellular changes that trigger hyperpigmentation through the activation of pathways regulated by epigenetic modifications. However, limiting or decreasing DNA methylation through sunscreen, niacinamide, and retinoic acid treatments that provide photoprotection and genetic transcription can counteract this.

## 1. Introduction

Epigenetics is the study of chemical modifications of the genome that do not affect cellular DNA sequences in a heritable and reversible way [1]. The most characteristic epigenetic change is the DNA methylation that consists of transferring a methyl group from methyl donors to cytosine residues from CpG islands in 5′ positions [2]. This transfer is regulated by DNA methyltransferases (DNMTs), such as DNMT1, DNMT3a, and DNMT3b. DNMT1 is expressed through encoding that maintains genome methylation during normal cell cycles, and is involved in the repair of damaged DNA [3]. DNMT3 encodes the novo DNA methylation process that occurs when cells initiate differentiation. DNMT3b has a higher expression than the DNMT3a variant and is mainly expressed in the basal layer of human skin [4]. Methylation-related changes in genomes are relevant to research that involves environmental factors [5]. Melasma is a common, acquired pigmentary disorder characterized by chronic and relapsing hypermelanosis on sun-exposed areas causing emotional and psychosocial distress [6]. It is more prevalent among Latin American and Asian women with darker skin phototypes [7]. Although epigenetic variations in melasma have not been documented, this condition shows alterations in the nuclear micromorphology from basal keratinocytes, which justifies its investigation [8]. These epigenetic variations include an increased diameter, perimeter, and texture of chromatin, measured by digital analysis and volumetric box-count methods [9]. These variations may be related to a greater amount of methylated DNA, which may induce the expression and activity of proinflammatory or promelanogenic genes [10].

In countering this DNA-cell damage, niacinamide and retinoic acid are two nonprimary depigmenting agents that can induce DNA-demethylation by decreasing the activity of DNMTs. Niacinamide enhances DNA repair, probably by preventing ultraviolet-induced ATP depletion [11]. This, along with its anti-inflammatory and melanosome transference blockage, can result in melasma improvement [12]. Meanwhile, retinoic acid enhances epidermal turnover and inhibits melanosome transference, facilitating the penetration of depigmenting medications [13]. It aids in the DNA methylation of* in vitro* cells, so it may also be beneficial to those with melasma [14]. In relation to photoprotection, it is well recognized that sunscreen reduces the severity of melasma [15]. This may reduce the genotoxic action of solar ultraviolet radiation on skin cells that prevents DNMTs activity and further genome methylation [16]. Therefore, in this study we sought to quantify the expression and activity of DNMTs that affect the basal conditions of DNA methylation in skin with malar melasma, and their modification following its treatment with sunscreen in combination with niacinamide, retinoic acid, or placebo.

## 2. Materials and Methods

### 2.1. Patients

Female patients having had malar melasma for more than two years and having attended our outpatient clinic between 2016 and 2017 were invited to participate in our study. Inclusion criteria were healthy volunteers 18 years of age and older, those without any previous treatment and without any photoprotection measures within the previous four weeks, and those who had Melasma Activity and Severity Index (MASI) scores higher than seven. Exclusion criteria were pregnancy or nursing, menopause, the coexistence of other pigmentation conditions, heat exposure, regular exercise, diet restrictions, consumption of food supplements or any type of drugs (including anti-inflammatories and hormonal treatments) within the previous two months, and having had given birth within a year. All subjects gave informed consent before providing a sample collection. This study was approved by our institution's ethics committee and was registered at the United States National Institutes of Health Clinical Trial Register (No. NCT03392623).

### 2.2. Study Design

The investigation was an eight-week, randomized, double-blind, controlled trial study conducted in the Dermatology Department of the Hospital Central Dr. Ignacio Morones Prieto of San Luis Potosí, México. Clinical data collected included age, skin phototype, disease duration, family history of melasma, occupation, solar habits, common triggering factors, and MASI scores. The patients were randomly allocated by permuted blocks into three groups of ten patients each. All received a 50+ SPF broad-spectrum sunscreen containing benzophenone-3, octinoxate, octocrylene, titanium dioxide, zinc oxide, and iron oxide as active ingredients [15]. In a double-blind, each group received 4% niacinamide, 0.05% retinoic acid (Valeant, NJ, USA), or a placebo in the form of Cetaphil® moisturizing cream (Galderma, Ontario, Canada). Niacinamide was formulated by combining niacinamide powder (Spectrum, Gardena, CA) with the aforementioned moisturizing cream [17]. Sunscreen was indicated every four hours during the day, and an application of one of the types of treatment was applied every night onto affected areas of the face. Skin pigmentation was objectively measured using a reflectance spectrophotometer (Chromameter CR-300, Minolta, Osaka, Japan). At each visit, coloration changes were examined using the luminosity (L*∗*) scale, for scores of 0 (full black) to 100 (total white), and the erythema (a*∗*) axis, for scores of 0–50. Pigment improvement was assessed on an L*∗* axis, with a score based on the average of six measurements of the affected areas. Differences were assessed by comparing initial and final L*∗* and a*∗* axis scores.

### 2.3. Skin Specimens

Under local anesthesia, two 3 mm punch biopsies, 10 mm apart, from lesional and adjacent nonlesional facial skin, were performed. Tissues for immunofluorescence and reverse transcriptase real-time polymerase chain-reaction (RT-qPCR) analyses were stored frozen in sterile tubes at minus 80°C until they were processed.

### 2.4. Global Methylation State

Frozen skin sections (3 mm thick) from lesional and healthy adjacent skin were processed for immunofluorescence. Skin sections of 4 *μ*m thickness were fixed with 10% Paraformaldehyde. Then, the sections were permeabilized with 0.1% Triton X-100 for 30 minutes, 4N HCl for 10 minutes, BTHCL for 15 minutes, and 0.05% Tween 20 overnight; we washed the sections with a phosphate-buffered saline solution (PBS) for 10 minutes between each application of permeabilization solution. Slides were incubated with a blocking reagent, diluted in a phosphate-buffered saline solution for 20 minutes, washed with PBS, and then incubated with a primary antibody (1:100) anti-human 5-methylcytosine monoclonal mouse (sc-56615, Santa Cruz Biotechnology, CA, USA) for 3 hours in a humidity chamber at room temperature in order to detect the methylcytosine from CpG islands. The sections were then washed with PBS, incubated for 2 hours with a secondary antibody (1:80) Goat anti-mouse IgG Alexa Fluor 594 (Thermo Fisher Scientific, MA, USA), and subsequently counterstained with a 4′ 6-diamidino-2-phenylindole (DAPI) (Thermo Fisher Scientific) for 10 minutes. Finally, the sections were treated with ProLong™ Gold Antifade Mountant and stored until being analyzed in a fluorescence microscopy AmScope FM800T series with a bandpass filter of 520 nm equipped with an INFINITY3 digital camera (Lumenera Corporation, Ottawa, Canada). The resulting images were analyzed with INFINITY ANALYZE version 6.5.2 software (Lumenera Corporation) without postacquisition processing. A fluorescence histogram was used for each sample, and the number of methylated cytosines was measured in fluorescence units.

### 2.5. Sample Processing for Reverse Transcriptase Real-Time PCR

Skin biopsies were mechanically lysed with liquid nitrogen, and total ribonucleic acid (RNA) was extracted using a phenol-chloroform gradient from each sample. Complementary deoxyribonucleic acid (cDNA) was synthesized from 100 ng of total RNA in a volume of 10 mL using the first-strand cDNA synthesis SuperScript II kit (Invitrogen, Carlsbad, CA) and oligo (dT)18 as primers (Invitrogen) in accordance with the manufacturer's recommendations. The cDNA concentration for all samples was adjusted to 100 ng/*μ*L. Real-time PCR was conducted in a MiniOpticon™ real-time PCR (Bio-Rad S.A., Hercules, CA). We used 10 *μ*L reaction volumes containing 1 *μ*L cDNA, 5 *μ*L iTaq™ Universal SYBR® Green Supermix (Bio-Rad), 0.2 *μ*L of each primer 20 mM (Integrated DNA Technologies, Inc, San Jose, CA), and 3.6 mL H_2_0 DEPC. The primers sequences were as follows:* DNMT1*, forward 5′-ggactcgttcagaaagccca-3 and reverse 5′-ctctggttgcgtgttgttgg-3′;* DNMT3b*, forward 5′-cggaagggagaatatgacag-3′ and reverse 5′-tgaagccagtgaacctcctct-3′. As endogenous control, we used* 18s*, forward 5′-cggctaccacatccaaggaa-3′ and reverse 5′-gctggaattaccgcggct-3′. The results were obtained by normalizing each sample with respect to its own endogenous control (*18s* gen). The normalized expression = gen problem/18s.

### 2.6. Statistical Analyses

We estimated that a sample size of ten patients would detect a difference in the expression of any DNMTs between treatment and control of 0.3 (i.e., 0.6 for niacinamide and retinoic acid and 0.3 for placebo), assuming an SD of 10, at 95% CI, and two tails, with *α* of 0.05 and *β* of 0.9. Therefore, 30 subjects with melasma were needed, given the three types of treatment applied. A descriptive analysis of the demographic variables of the study subjects was carried out through measures of central tendency, as well as an analysis of MASI scores and colorimetric values, before and after treatment. For the comparison of DNMT1 and DNMT3b expressions in melasma-affected skin and unaffected skin, we used a Student's t-test. One-way ANOVA and post hoc Tukey were used to compare the levels of methylated DNA following treatments with sunscreen in combination with niacinamide or retinoic acid to the levels with sunscreen in combination with placebo. All values were set to a confidence level of 95%, considering a significant p of 0.05.

## 3. Results

Of the 274 female patients assessed with malar melasma, 30 were included for this study. As shown in Table 1, the groups they were put into did not differ in demographics, clinical features, or melasma history. All the patients showed a clinical improvement of their condition after treatment. The 4% niacinamide group showed the most improvement in MASI scores and colorimetric values, followed by the 0.05 retinoic acid group and then the placebo group.  Table 2 compares basal and posttreatment data in the form of MASI scores and colorimetric values.

### 3.1. Global Methylation in Melasma Skin and Perilesional Skin

**Figure 1:**
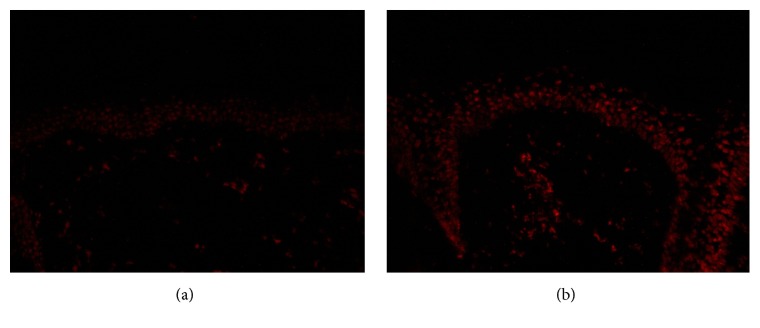
Epidermal presence of 5-methylcytosine. Representative comparison of indirect immunofluorescences of skin biopsies from melasma patients. The unaffected skin exhibits a decreased fluorescence (a), and melasma skin shows a bright fluorescence in dermis and epidermis (b) (x400).

**Figure 2:**
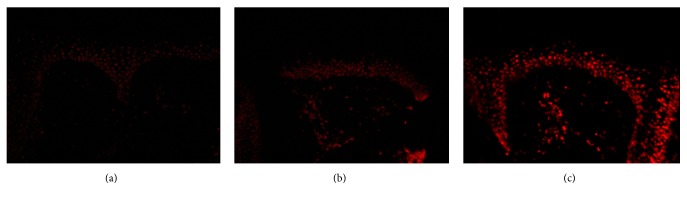
Epidermal presence of 5-methylcytosine after treatment. Representative comparison of indirect immunofluorescences of melasma skin after 8 weeks of treatment. Treated skin with 4% niacinamide (a) and 0.05% retinoic acid (b) show lower specific staining than placebo (c) (x400).

All melasma skin samples exhibited significantly augmented fluorescence, both in epidermis and dermis, compared to nonlesional skin before treatment (Figure 1). Globally, lesional skin showed a greater fluorescence than unaffected skin (71 ± 5 versus 55 ± 5; t-test,* P* < 0.001). Compared with basal values, at the end of the trial there was a significant reduction of specific staining in melasma lesions in the niacinamide group (70 ± 5 versus 50 ± 3;* P* = 0.04) and the retinoic acid group (74 ± 2 versus 53 ± 5; P = 0.04), both in epidermis and dermis; however, the placebo group showed no such reduction (68 ± 3 versus 64 ± 2;* P* = 0.4). The niacinamide and retinoic acid groups also showed a lower staining compared to the placebo group (ANOVA,* P* < 0.001). These data are included in Table 2, and representative images are shown in Figure 2.

### 3.2. Expression of DNA Methyl Transferases in Melasma Skin and Perilesional Skin

**Figure 3:**
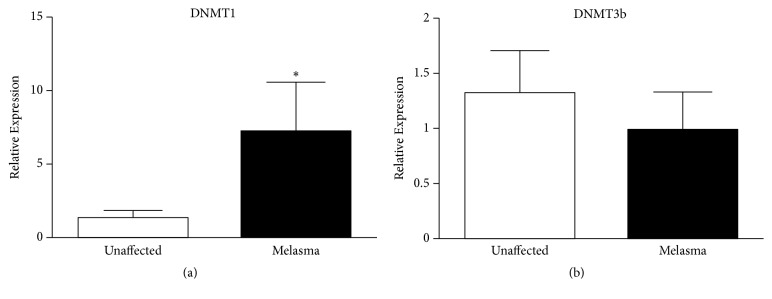
Expression of DNA methyltransferases in melasma patients. Relative expression of DNMT1 (a) and DNMT3b (b) mRNAs in skin biopsies of unaffected and melasma skin by RT-qPCR. The results were normalized with respect to* 18s* gen. The results correspond to the mean ± SD of 30 patients at basal conditions. *∗* Significant differences with p<0.05 (P values for t-test).

**Figure 4:**
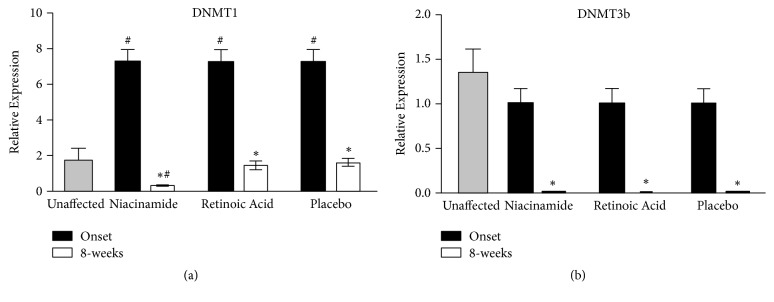
Expression of DNA methyltransferases after interventions. Relative expression of (a) DNMT1 and (b) DNMT3b mRNAs in skin biopsies from patients at basal and after 8 weeks of treatment with sunscreen, 4% niacinamide, and 0.05% retinoic acid quantified by RT-qPCR. The results were normalized with respect to* 18s* gen. The results correspond to the mean ± SD of each group (n=10). *∗* Significant differences before treatment, # significant differences with respect to unaffected skin, with p<0.05 (P values for ANOVA with post hoc Tukey test).

Prior to treatment, the gene expression of DNMT1 was significantly increased in melasma lesions compared to nonlesional skin (t-test* P =* 0.02), as depicted in Figure 3. After eight weeks of treatment, a significant decrease of DNMT1 was observed in melasma lesions in all groups (ANOVA,* p = 0.02*). Lesions treated with 4% niacinamide showed the greatest reduction. All three treatment types resulted in DNMT1 levels lower than those in unaffected skin (Figure 4(a)).

In relation to DNMT3b, the basal conditions of melasma lesions did not show any difference compared to unaffected skin (Figure 3); however, after treatment, DNMT3b expression diminished in all groups compared to basal conditions (ANOVA,* P * < 0.001), showing levels lower than those in normal skin (Figure 4(b)).

## 4. Discussion

Melasma is a common, acquired pigmentary disorder characterized by chronic and relapsing hypermelanosis of sun-exposed areas [6]. Its etiopathogenesis is complex and includes diverse networks of cellular interactions between melanocytes, keratinocytes, Langerhans cells, mast cells, extracellular matrices, and cytokines involved in inflammation, production of growth factors, and hormonal stimuli [18, 19]. Environmental stressors such as solar radiation are the most important factors in triggering melasma. Gene-environment interactions involve mechanisms of response that include DNA methylation, synthesis of microRNAs, and histone modifications [2]. The main epigenetic change induced by environment is cellular DNA hypermethylation promoted by DNMT1, DNMT3a, and DNMT3b [4]. This phenomenon induces heterochromatin with lower gene-transcriptional activity [20]. These changes are supposed in melasma by the presence of chromatin variations in keratinocytes, observed through histologic studies and fractal-dimension image techniques [9].

At the end of treatment, all three groups showed improvement in melasma and reduction in DNMT1 and DNMT3b. Concerning the DNA methylation of melasma, niacinamide and retinoic acid, but not placebo, improved this condition. Possible explanations are that niacinamide may be exerting a DNA-repairing function since it is a precursor of nicotinamide adenine dinucleotide (NAD +) and its phosphorylated form (NADP+). Both NAD+ and NADP+ are substrates for poly-polymerases, which are involved in any response to DNA damage, including the repair and maintenance of genomic stability [21]. Retinoic acid may be reducing the methylation status by regulating the activation of the RET gene. This gene activates repressor complexes which blockade the enhancers' complexes that initiate the methylation process [14]. The lack of methylation improvement in the placebo group may be due to the fact that sunscreen does not contain active ingredients capable of removing the methyl groups already present within damaged DNA.

The association between DNA hypermethylation and hyperpigmentation may be related to the inhibition and repression of hypermethylated genes [1]. The WnT pathway activates genes implicated in pigmentation, and it is regulated by gene methylation [22]. The hypermethylation of WnT repressors enhances the differentiation and proliferation of melanocyte stem cells [23]. Therefore, the depigmenting actions of niacinamide and retinoic acid may be related to the upregulation of the expression of the secreted frizzled-related protein-1 (SFRP1), a repressor of the WnT/*β*-catenin pathway that inhibits Tyrosinase and MITF transcription decreasing the melanin synthesis [24].

The greater expression and activity of DNMT1 found in melasma lesions than in unaffected skin is probably related to solar exposure. Previous reports had shown that ultraviolet exposure increases the DNMTs expression, as they are also elevated in photodamaged skin [16]. Thus, the augmented expression may be related to a damaged microenvironmental skin with an associated DNA hypermethylation status [16]. On the other hand, DNMT3b expression in melasma was similar to unaffected skin, but it was reduced after eight weeks of treatment in all three groups compared to its basal values. DNMT3b participates in DNA methylation mainly in the basal layer of the skin during skin stem-cell proliferation and differentiation [25]. The effect of treatments could explain the reduction found in both enzymes. Niacinamide and retinoic acid may be inhibiting them by downregulating the presence of reactive oxygen species (ROS) which are DNMTs activators [26, 27]; retinoic acid in addition may be activating miRNAs, such as miR-152 that decreases DNMTs [28]. The sunscreen use may be blocking the enzyme methylation genes induced by solar ultraviolet radiation conditioning its inhibition [16, 24]. Thus, the melasma improvement, accompanied by the reduction of both enzymes on different locations, implies that treatment acts on diverse layers of the epidermis.

Limitations in our study include a reduced population sample, the exclusive malar melasma type studied, and the fact that we studied the DNA of skin cells without considering cellular subgroups. However, our study helps to explain the mechanisms by which photoprotection and nonprimary depigmenting agents such as niacinamide and retinoic acid improve melasma [12, 15]. There is evidence that nonprimary depigmenting agents have positive effects on melasma and acquired hyperpigmentation [12, 29]. These agents may decrease inflammation, increase cellular turnover, and/or inhibit melanosome transference and melanocyte differentiation [12]. We found that reducing methylation in the melasma skin might be another mechanism to be considered in melasma. Therefore, combining actives that modulate cutaneous epigenetic processes along with depigmenting agents may render a synergic therapeutic outcome.

Thus, further research on the mechanisms of methylation in genes involved in the physiopathogenesis of melasma may provide more insights for specific treatments. Future perspectives will focus on the association of methylation with the activation of inflammatory genes involved in melasma [19]. Likewise, it will be interesting to evaluate other epigenetic mechanisms such as histone modifications and microRNAs in order to better quantify the intensity of cellular DNA damage and its repair using specific drugs. Additional investigations, including in vitro studies, would be necessary to discover the mechanism that induces the downregulation of DNMTs and the hypomethylation of melasma patients.

In conclusion, we provide the first evidence of the role of DNA methyltransferases in melasma. We observed that DNMT3 and DNMT1 are increased in melasma and downregulated by the use of sunscreen in combination with 0.05% retinoic acid, 4% niacinamide, or placebo. Therefore, DNA methylation may be involved in the process of augmented pigmentation found in melasma, and treatment aimed at reducing cellular DNA methylation states may reduce pigmentation and clinically improve its lesions.

## Figures and Tables

**Table 1:** Main demographic and clinical features of the 30 studied subjects treated with 4% niacinamide, 0.05% retinoic acid, or placebo over 8 weeks.

	4% Niacinamide group (n=10)	0.05% Retinoic acid group (n=10)	Placebo group (n=10)
Age (year; mean ± SD)	25 ± 3.3	28 ± 3.2	26 ± 1.5

Skin phototype n (%)			
IV	1 (10)	1 (10)	2 (20)
V	9 (90)	9 (90)	8 (80)

Duration of melasma (yr), mean ± SD	5.3 ± 3.3	3.8 ± 4.2	4.2 ± 4.3

Family history, *n *(%)	4 (40)	4 (40)	6 (60)

Occupation *n *(%)			
Housewife	10 (100)	10 (100)	7 (70)
Indoor worker	-	-	3 (30)

Predisposing factors, *n *(%)			
Sun exposure	10 (100)	10 (100)	10 (100)
Pregnancy	-	-	-
Oral intake of hormones	-	-	-
Artificial sources of radiation	2 (20)	-	3 (30)

There were no significant differences in age, skin phototypes, melasma duration, and other clinical features among groups.

**Table 2:** Changes in colorimetric values (L*∗*, a*∗*), MASI index, and Mean Fluorescence Intensity (MFI) in melasma lesions treated with 4% niacinamide, 0.05% retinoic acid, and placebo; initially and at the end of the study.

	Initial values				Values after 8 weeks of treatment			
	4% Niacinamide (n=10)	0.05% Retinoic acid (n=10)	Placebo (n=10)	P value	4% Niacinamide (n=10)	0.05% Retinoic acid (n=10)	Placebo (n=10)	P value

L*∗*	46.5 ± 3.8	47.2 ± 3.2	47.7 ± 3.8	0.16	52.7 ± 2.3**∗∗**	50.2 ± 3.1	48.4 ± 4.2	0.01

a*∗*	11.7 ± 1.8	12.1 ± 0.5	12.7 ± 0.9	0.1	11.4 ± 1.2	12.3 ± 1.8	12.6 ± 2.5	0.06

MASI	15.4 ± 6.7	18.5± 13.5	9.1± 1.4	0.1	10.4 ± 5.1	12.9 ± 6.4	7.1 ± 1.2**∗∗**	0.03

MFI	70 ± 5	74 ± 2	68 ± 3	0.4	50 ± 3	53 ± 5	64 ± 2**∗∗**	<0.01

L*∗*: axis values; a*∗*: erythema axis values; p values obtained by *t *test. The groups did not differ in colorimetric, MASI, and MFI values at onset (ANOVA, P >0.05).

## Data Availability

The data used to support the findings of this study are restricted by the Hospital Central Dr. Ignacio Morones Prieto Ethics Board in order to protect patient privacy. Data are available from Bertha Torres-Alvarez, MD, for researchers who meet the criteria for access to confidential data. The blinded data used to support the findings of this study are available from the corresponding author upon request.
